# Image annotation and curation in radiology: an overview for machine learning practitioners

**DOI:** 10.1186/s41747-023-00408-y

**Published:** 2024-02-06

**Authors:** Fabio Galbusera, Andrea Cina

**Affiliations:** 1 Spine Center, Schulthess Clinic, Lengghalde 2, Zurich, 8008 Switzerland; 2 ETH Zürich, Department of Health Sciences and Technologies, Zurich, Switzerland

**Keywords:** Artificial intelligence, Data curation, Image processing (computer-assisted), Machine learning, Privacy

## Abstract

“Garbage in, garbage out” summarises well the importance of high-quality data in machine learning and artificial intelligence. All data used to train and validate models should indeed be consistent, standardised, traceable, correctly annotated, and de-identified, considering local regulations. This narrative review presents a summary of the techniques that are used to ensure that all these requirements are fulfilled, with special emphasis on radiological imaging and freely available software solutions that can be directly employed by the interested researcher. Topics discussed include key imaging concepts, such as image resolution and pixel depth; file formats for medical image data storage; free software solutions for medical image processing; anonymisation and pseudonymisation to protect patient privacy, including compliance with regulations such as the Regulation (EU) 2016/679 “General Data Protection Regulation” (GDPR) and the 1996 United States Act of Congress “Health Insurance Portability and Accountability Act” (HIPAA); methods to eliminate patient-identifying features within images, like facial structures; free and commercial tools for image annotation; and techniques for data harmonisation and normalisation.

**Relevance statement** This review provides an overview of the methods and tools that can be used to ensure high-quality data for machine learning and artificial intelligence applications in radiology.

**Key points**

• High-quality datasets are essential for reliable artificial intelligence algorithms in medical imaging.

• Software tools like ImageJ and 3D Slicer aid in processing medical images for AI research.

• Anonymisation techniques protect patient privacy during dataset preparation.

• Machine learning models can accelerate image annotation, enhancing efficiency and accuracy.

• Data curation ensures dataset integrity, compliance, and quality for artificial intelligence development.

**Graphical Abstract:**

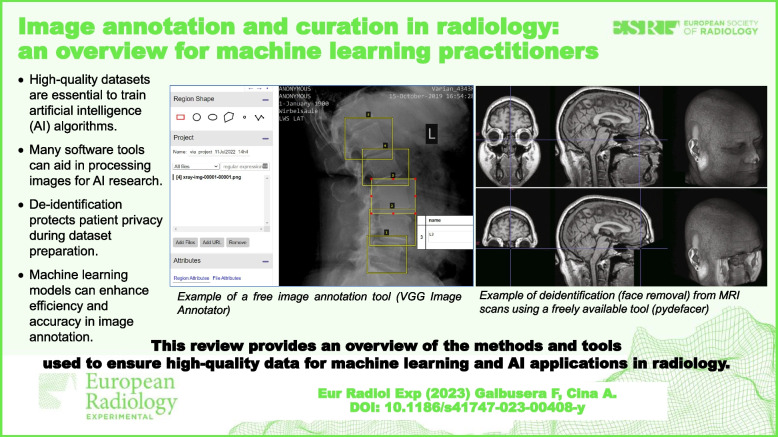

## Background

High-quality datasets have paramount importance in artificial intelligence (AI). “Garbage in, garbage out” is indeed a universally recognised principle not only of machine learning (ML) but of computing in general. All tasks aimed at ensuring that the data used to train and validate AI-based algorithms are consistent, standardised, traceable, and correctly annotated therefore constitute a critical part of the development of valid, reliable, and robust tools.

A bottleneck for the creation of large databases to train AI models is the time-consuming task of image annotation. In fact, hospitals have large quantities of images available that cannot be used to train supervised learning models due to the lack of annotation. In the last years, many works focused on the development of image annotation tools to speed up the process [1–3].

An important role during database creation is played by the anonymisation process to comply with the privacy regulations. The anonymisation can be done manually, but some pipelines have been proposed to automatise and speed up the process [4]. Some of the presented tools have been embedded in web applications that can be also easily used by healthcare professionals without a technical background.

This narrative review aims to describe different aspects related to the creation and use of medical imaging databases in the ML domain. The focus is on the need to standardise image curation and annotation, which would allow for example the implementation of “federated learning”, a paradigm that seeks to address the problem of data governance and privacy by training algorithms without exchanging the data itself [5]. Indeed, interoperability among hospitals is prevented because of different ways to store data. To overcome this problem, there is some research trying to address the interoperability of image annotation [6].

After a brief introduction to medical image formats, basic image processing concepts, and software that can be used to work with such images, this narrative review describes the cornerstones of generating a high-quality imaging dataset, namely de-identification, annotation, and curation, with special emphasis on free software that can be readily used for this purpose by interested researchers. The key information, namely the steps to generate a radiological dataset to be used for developing ML applications, is summarised in Table 1. The paper is addressed to all healthcare professionals both with and without a technical background; engineers working in healthcare have an overview of the state-of-the-art methods from which they can start to improve existing tools while clinicians can become aware of the different tools and they can use to automatise some routine processes.

**Table 1:** Workflow to create a dataset of annotated images to be used for machine learning applications

**Step**	**Description**
Definition of the data of interest	Specifying what kind of data should be collected and annotated for the project, in terms of imaging modality, protocol, anatomy, pathology, and clinical question that are relevant for the application.
Data collection and de-identification	Acquiring the imaging data from the source, such as a PACS system, a DICOM server, or a public repository. The data should be representative of the target population and environment. Data must be de-identified by removing any personal or sensitive information that can identify the patients or the institutions. Compliance with the ethical and legal regulations, such as HIPAA or GDPR, must be ensured.
Annotation	Labelling the data with the information that is needed for the machine learning task, such as bounding boxes, polygons, masks, or tags. A standard protocol or guideline for annotation should be followed. The annotation must be accurate, consistent, and complete. Either custom computer programs or existing software, free or proprietary, may be used to facilitate this process.
Curation	Reviewing and validating the annotated data and resolving any errors or discrepancies. Multiple experts or consensus methods to check the quality and reliability of the annotation may be employed. Software tools can be used to manage and monitor the annotation process.
Storage	Storing and organising the annotated data in a format that is suitable for machine learning, such as DICOM, NIfTI, or PNG. Data must be secure and accessible for the machine learning framework and model. Specific software tools can be employed to track and version the data.

## Image formats

Medical images may be of different types. A medical image dataset can be made of images representing the projection of an anatomical volume onto an imaging plane (two-dimensional [2D] image), a series of images representing thin slices through a volume (each slice is 2D, and they constitute a three-dimensional [3D] volume), a series of data from a volume (3D), or multiple acquisitions of the same tomographic or volumetric image over time to produce a dynamic series of acquisitions (four-dimensional [4D]) [7]. The reference format for storing and transmitting medical images in healthcare institutions is “Digital Imaging and Communications in Medicine” (DICOM). DICOM has been first released in the 1980s and has become a standard globally accepted and employed by vendors of imaging equipment and IT systems, including Picture Archiving and Communication Systems (PACS) which are the foundation of imaging databases in almost every hospital [8]. Due to its backward compatibility, which has been implemented since 1993, the use of DICOM ensures interoperability between any equipment and storage solutions, either modern or more dated, or marketed by different manufacturers.

A DICOM object consists of a set of attributes (patient name, identifier, study date, etc.). Pixel data, *i.e.*, the image itself, is itself an attribute, while each object can contain only a one-pixel data attribute, such an attribute can include various “frames”, practically allowing for storing multi-frame data in a single file. The pixel data attribute can be uncompressed or compressed, in the latter case either losslessly or with an algorithm such as the Joint Photographic Experts Group (JPEG) format.

Although DICOM is the de facto standard used by almost every vendor, other file formats are widely used in medical imaging, especially among researchers and AI developers. Since backward compatibility is not mandatory for such applications, more modern formats, allowing for example for easier management of a whole series of images in a single file, have found a place in the field. Well-known examples of such formats include the Neuroimaging Informatics Technology Initiative (NIfTI) (https://nifti.nimh.nih.gov/), Analyze, and the Whole Slide Image (WSI) format [9]. The main difference between DICOM and NIfTI/Analyze is that the raw image data in the latter formats are saved as a 3D image, while in DICOM, they are saved as 2D image slices [10]. Besides, Analyze and NIfTI have a simpler structure in comparison with DICOM and are easier to parse. As a matter of fact, DICOM is not very efficient by current standards and remains the dominant format for clinical applications only due to the backward compatibility issue. The NIfTI format is mainly used for neuroimaging since it was developed in that field, but it can be effectively employed for any medical applications [11]. Analyze files are usually stored as a 2-item file where one item contains the pixel information and the other item the metadata [7], while NIfTI contains all information in a single file.

DICOM and NIfTI support compression and encryption, while Analyze does not. The WSI file format stores the high-resolution histological image in a pyramidal file where we can access the same image at different resolutions. Moreover, as for DICOM files, WSI also contains metadata. WSI was developed specifically for digital pathology limiting its actual use to a very specific domain [12]. Computer languages used for ML applications such as “Python” (https://www.python.org/) and “R” (https://www.r-project.org/) provide libraries to read and write these file formats, as well as DICOM files. Standard image formats not developed for medical use such as Tagged Image File Format (TIFF), Bitmap Image File (BMP), and Portable Network Graphics (PNG) are also commonly used in research, typically in conjunction with files containing metadata in plain text or standard formats such as eXtensible Markup Language (XML, https://www.w3.org/TR/REC-xml/) and JavaScript Object Notation (JSON, https://www.json.org/).

## Imaging key concepts

Medical images have a set of features that should be considered when they are processed: the pixel depth, the photometric interpretation, the metadata, and the pixel data. The latter two have already been mentioned in the previous paragraph and will therefore not be detailed further.

Pixel depth indicates how many bits are used to display the information of a single pixel. A bit is the smallest building block of digital information, and it can assume only two values, namely 0 or 1. In general, an image can be displayed with *2*^*n*^* − 1* intensity levels where *n* is the number of bits. For a binary image (only black and white), we need, for each pixel, only 1 bit that can have a value of 0 (black) or 1 (white). Grayscale images are usually represented as 8-bit images, and so, the pixels can assume values from 0 (black) to 255 (white); 12-bit or 16-bit pixel depths are also common in medical imaging [13]. The photometric interpretation indicates if the pixel data should be interpreted as a colour or a grayscale image.

X-rays, computed tomography (CT), and magnetic resonance imaging (MRI) images are usually stored as grayscale images where each pixel has a unique value. Positron emission tomography and single-photon emission tomography are typically displayed with a colour map, but the pixel information has only one value as for the previously presented images. These images are said to be in pseudo-colour [7]. To have true colour images, the machine acquires more samples for each pixel, and using colour models [14] converts the samples into colours. A typical colour model is the “red, green, and blue” (RGB), where the pixel that is displayed should be considered as a combination of the three primary colours, and so three samples for each pixel are stored [15]. Each pixel sample belongs to a so-called channel, and in fact, colour images are known as 3-channel images. Usually, RGB images, such as for example ultrasound images, are referred to as 24-bit colour images (8-bit for each channel). An example of RGB images is ultrasound images. The metadata, in addition to the set of attributes presented in the previous paragraph, contain information about the image acquisition and the image features such as resolution, pixel depth, and photometric information. Moreover, metadata can include data about radiologists’ annotations that can be used both for research and for patient retrieval in clinical studies. Finally, as explained before, the pixel data stores the image itself, and so, it contains the matrix with the numbers representing the pixel intensities.

## Free software for medical imaging

The scientific research community has always shown great interest in free software and substantially contributed to the development of publicly available software for several applications. Image processing, including tasks that are more relevant for AI research such as anonymisation, curation, segmentation, and annotation of medical images, is no exception. As a matter of fact, AI developers can directly access, and modify and redistribute, high-quality software made available on university repositories or cloud services such as Sourceforge (https://sourceforge.net/) and GitHub (https://github.com/).

**Fig. 1:**
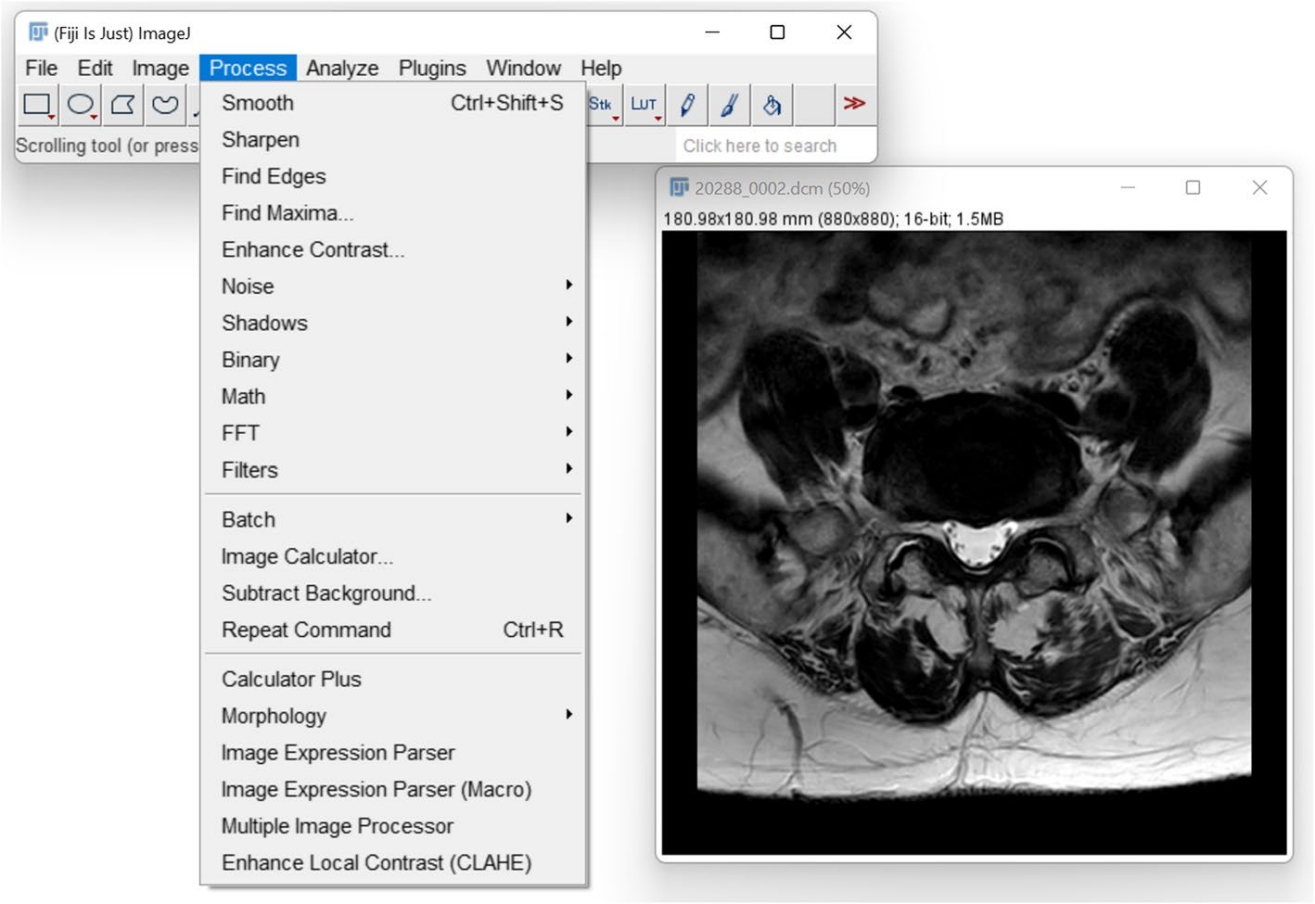
A screenshot of Fiji, a version of ImageJ packaged with several plugins

ImageJ [16, 17] (https://imagej.nih.gov/ij/) is a multiplatform general-purpose software for image processing and analysis written in Java. It is freely available in the public domain, with no licence required. It supports a large variety of file formats including all those used in medical imaging, either in its native version or through widely available plugins. Indeed, ImageJ has been used as a development platform for advanced image processing algorithms taking advantage of its extensible nature; hundreds of plugins have been made available by various academic developers, many of which are conveniently bundled in a single downloadable package, Fiji [18] (https://imagej.net/software/fiji/) (Fig. 1). Besides plugin-based extensions, ImageJ offers several tools to perform operations on images such as custom filtering, edge detection, sharpening, and geometric transformation, as well as analysis tools such as calculation of areas, distances, angles, and descriptive statistics on the value of selected areas. ImageJ directly supports multidimensional data such as image stacks from CT or MRI.

**Fig. 2:**
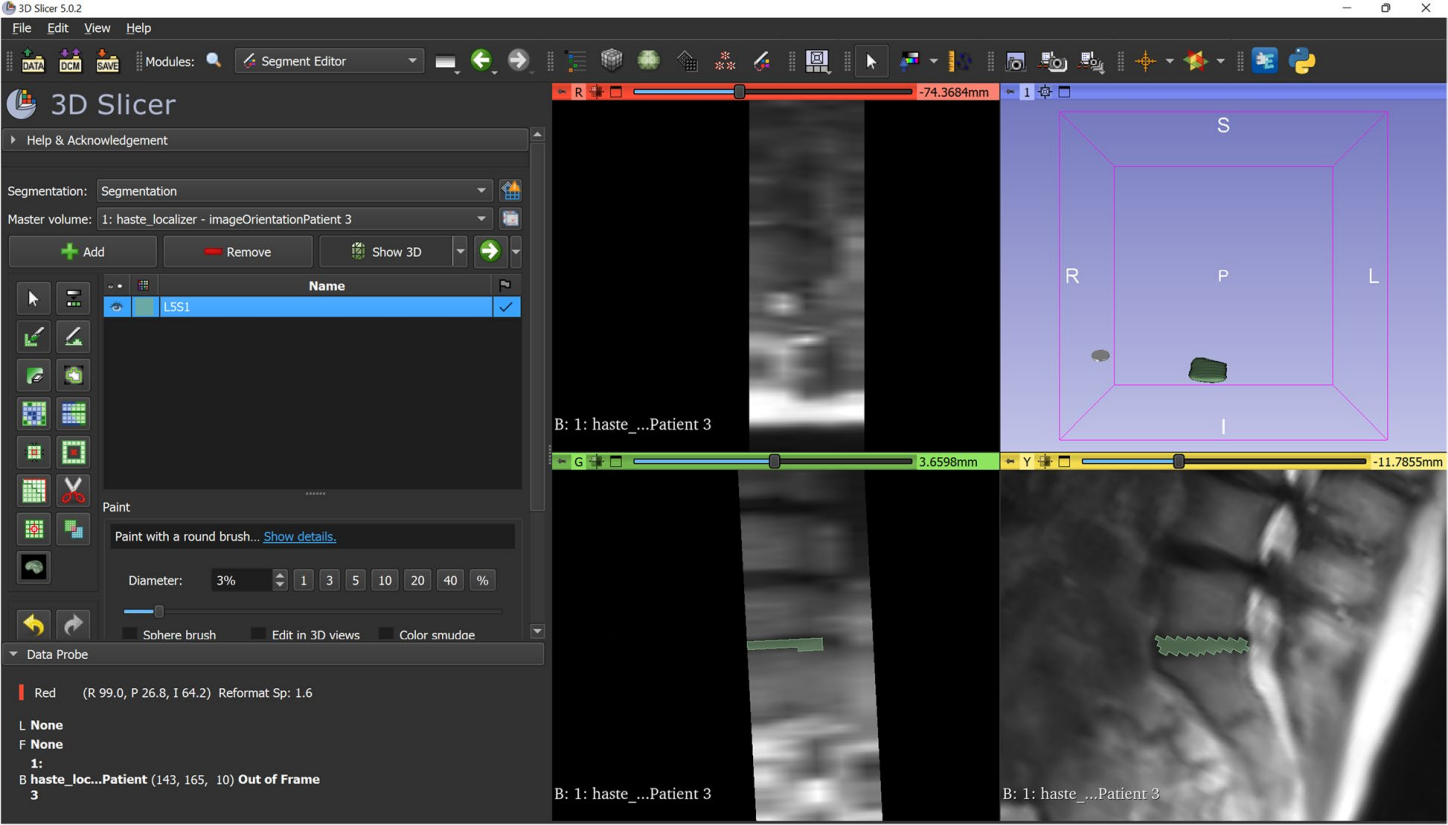
3D Slicer, a free software package for medical image processing and analysis

3D Slicer [19] (https://www.slicer.org/) is a software package offering advanced features for image processing with a focus on medical imaging. It provides support for multidimensional data in various formats including DICOM, as well as capabilities such as manual and semiautomatic image segmentation, image registration, advanced visualisation and rendering, measurement of areas, distances and angles, 3D printing, and virtual reality support (Fig. 2). 3D Slicer has a modular, extensible structure that has been exploited by its large community of users to develop tools for AI-based segmentation and surgical planning. Thanks to its features, user-friendly interface, and scripting capabilities, it is one of the most employed solutions to generate ground truth data for the development of AI tools for image processing.

**Fig. 3:**
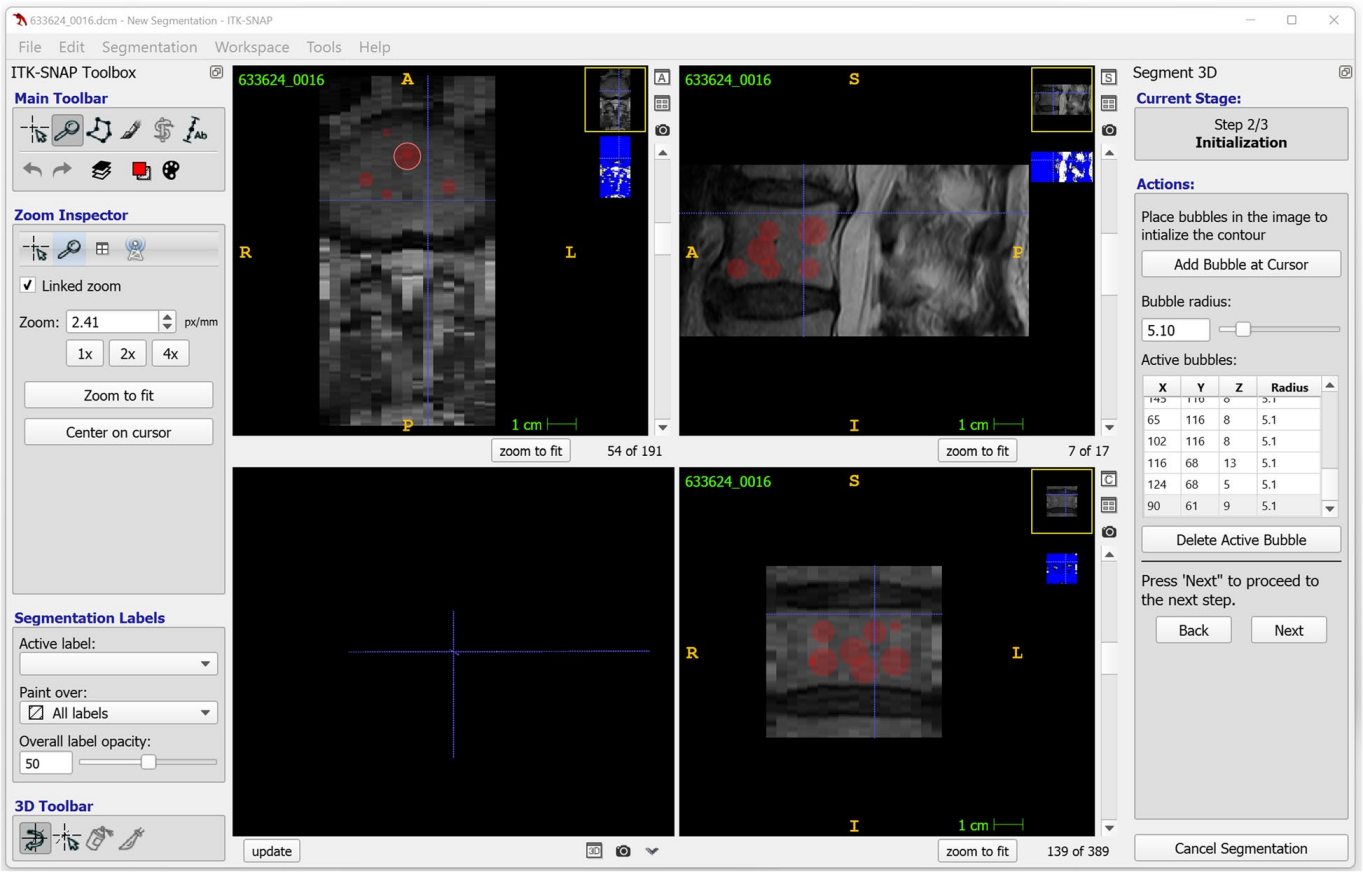
ITK-Snap, a tool for manual and semi-automatic image segmentation using active contours

ITK-Snap [20] (http://www.itksnap.org/) is a free software application based on the Insight Toolkit (ITK) (https://itk.org/) aimed at providing user-friendly tools for manual and computer-assisted image segmentation (Fig. 3). ITK-Snap supports all file formats commonly employed in medical imaging and offers a multi-window interface facilitating visualisation and interaction with the images. The built-in semi-automatic tool is based on active contours methods; in addition, recent versions of ITK-Snap offer a registration feature to improve the management of multimodal images, as well as a distributed segmentation service aimed at allowing cloud-based segmentation through algorithms provided by the developer community on the Internet.

Several other free software packages and libraries have been developed for medical image processing, such as for example Icy (https://icy.bioimageanalysis.org/) and DIPlib (https://diplib.org/), and are not discussed in this paper for the sake of brevity.

## Anonymisation and pseudonymisation

Medical images used for the development and validation of AI-based tools are typically de-identified, *i.e.*, they do not contain any type of information which can lead to an identification of the patient. Deidentification aims at preserving the privacy of the patient, in turn guaranteeing dignity, respect, and individuality [21, 22]. Besides, the use of de-identified data in medical research favours effective communication and trust in the relationship between patient and physician, with direct consequences on the quality of the provided healthcare as well as of the collected data.

Image deidentification is strictly governed by local, national, and supranational regulations, such as the General Data Protection Regulation (GDPR) in the European Union and the HIPAA (Health Insurance Portability and Accountability Act) in the USA. Depending on the specific research project or clinical trial, pseudonymisation may be employed instead of anonymisation; in the former case, the patient’s identifiable information is replaced with artificial identifiers, *i.e.*, pseudonyms, and such de-identified information is used for data analysis and processing [23]. By keeping track of the pseudonyms, this approach allows for restoring the original information at a later stage. In contrast, in full anonymisation, any link between personally identifiable information and the patient’s data and images is irrevocably lost. The process of anonymisation can involve several steps including removing identifiers, generalising data [24] (for example, rounding ages to the nearest decade), aggregating data (for example, stratify patients in age ranges), and further randomisation (shuffling data not useful to the analysis to make correlation impossible) [25].

One fundamental aspect is obtaining patient consent for data usage. GDPR and HIPAA both emphasise the need for informed and voluntary patient consent before any data collection or processing can occur. This consent ensures that patients are aware of how their data will be used, promoting transparency and trust between healthcare providers and patients [26]. Data encryption and access control are also important. Encrypting data ensures that even if unauthorised access occurs, the information remains unintelligible [27, 28]. Access controls limit who can view, edit, or delete data, ensuring that only authorised personnel have access to patient records.

Another critical aspect of data privacy regulations is the requirement for data retention periods. GDPR and HIPAA stipulate specific timeframes for how long patient data can be retained. This ensures that organisations do not keep data indefinitely, reducing the risk of unauthorised access over time [29, 30]. Both GDPR and HIPAA require the transparent reporting of data breaches. Organisations must inform authorities and affected individuals of any data breaches as soon as they are discovered. Failure to report breaches can result in severe legal consequences, including substantial fines and penalties [31]. These regulations are designed to hold healthcare organisations accountable for data breaches and to protect patient rights and privacy in an increasingly digital healthcare landscape. A common issue with the regulations is how much healthcare professionals know about them. A few studies investigated this issue, and the conclusion was that doctors and clinical researchers need to increase their knowledge about regulatory aspects [32, 33].

Going into details on how different data formats deal with anonymisation and pseudonymisation, the DICOM format includes attributes which are used for sensitive data allowing for patient identification; other formats such as NIfTI also include metadata which can be used for patient identification. Anonymising/pseudonymising images indeed means deleting such metadata, in the case of DICOM by erasing their content or by removing the attributes altogether. Attributes that should be deleted during de-identification include the patient’s name, address, and information about the hospital and referring physician.

Most PACS clients allow exporting anonymised DICOM files but do not typically offer specific control about how the de-identification is performed. In cases in which a finer control about de-anonymisation is needed, dedicated software should be used. Examples of free de-identification software include gdcmanon, which is part of the GDCM (Grassroots DICOM, http://gdcm.sourceforge.net/) tool collection to process DICOM files, and the Python package dicom-anonymizer (https://pypi.org/project/dicom-anonymizer/) which can be conveniently integrated into AI pipelines. Most of the dedicated de-anonymisation programmes are compliant with the DICOM standard; this is a critical aspect that needs to be considered, since an incorrect deletion of attributes may render the file not readable by viewers enforcing compliance to the standard.

**Fig. 4:**
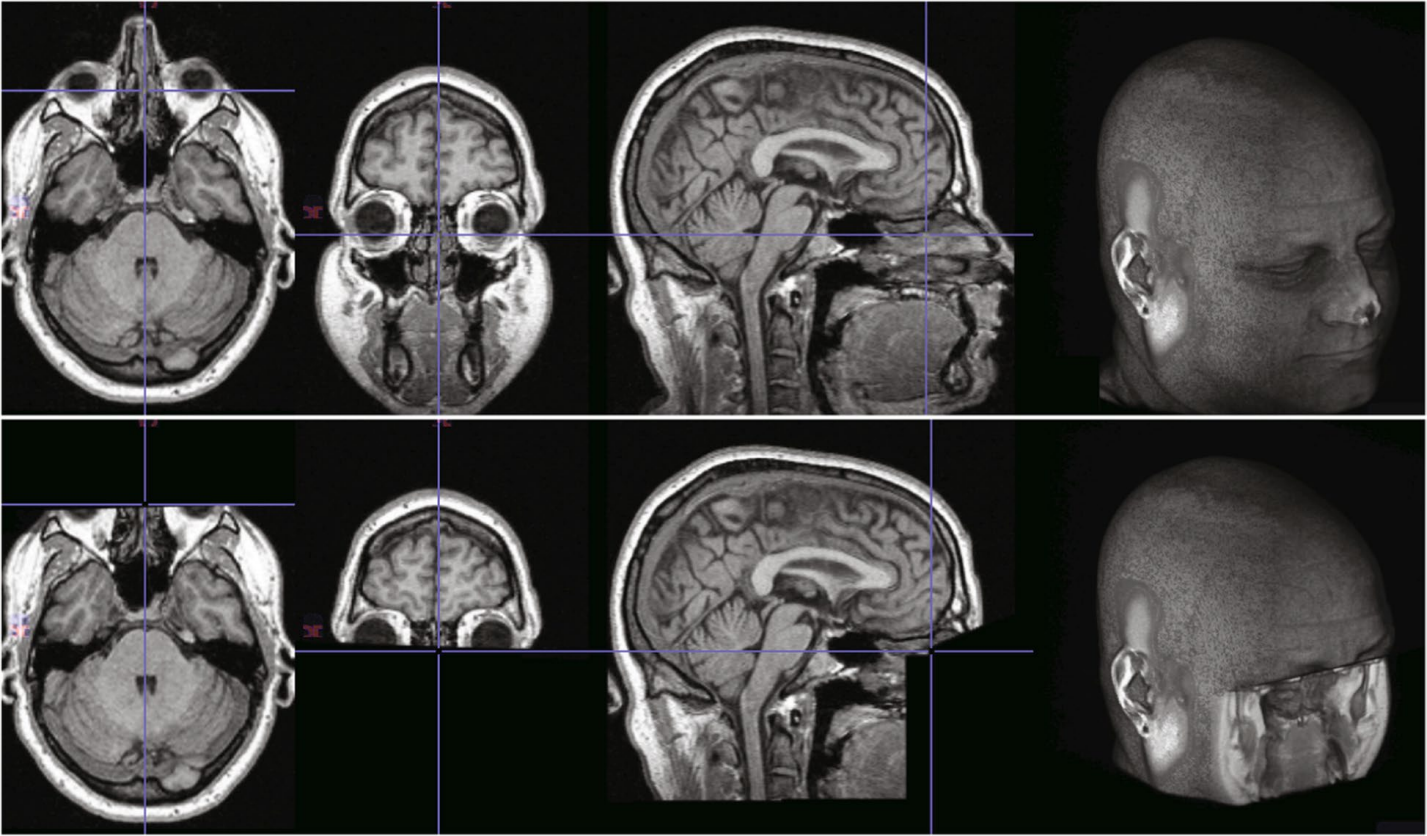
Example of face removal from MRI scans of the head obtained with “Pydefacer”. The first row depicts the original images, while the second shows the same images after the removal of the face. Reprinted from [22] (no permission required)

Besides the attributes containing personal identifiable information, the images themselves may allow the identification of the patient [34]. This is often the case for CT/MRI scans of the head containing facial features, which can potentially provide a recognizable 3D rendering of the patient. Several tools have been developed to automatically remove such facial features from scans, including the free Python library pydeface (https://github.com/poldracklab/pydeface) and the command line tool mridefacer (https://github.com/mih/mridefacer) (Fig. 4).

## Annotation

Supervised learning is based on the availability of labelled training data, *i.e.*, data with an associated “label”; developing an algorithm based on supervised learning indeed implies searching for a function able to map each training data point to the corresponding label. Labelled training data is also employed in self-supervised learning, typically as a second step after training a model on automatically generated pseudolabels [35].

In AI-based image processing, generating labelled training data involves associating information to each image, namely “annotations”. Typical annotations are as follows:

- Categorical variables describing the content of the image
- Segmentations
- Location and size of one or more regions of interest (each one potentially with a further label describing its content)
- Coordinates of landmarks

The categorical variable annotation is the most general as it requires to associate a single label to an image whether it is at image, exam, volume, or patient level. In this case, the need for a huge dataset is of paramount importance to be able to train the models on many different examples. However, in recent years, computer vision researchers started to work on new self-supervised techniques overcoming the limitations of small, annotated datasets. The application of self-supervised learning in the medical domain is limited, but it can grow in the future. The annotation process for segmentation or localisation tasks is more specific, and it requires more time. Therefore, generating annotation masks and landmarks for images can be a tedious task requiring significant time and manual labour, especially in the case of large sets of thousands, or even millions, of images.

**Fig. 5:**
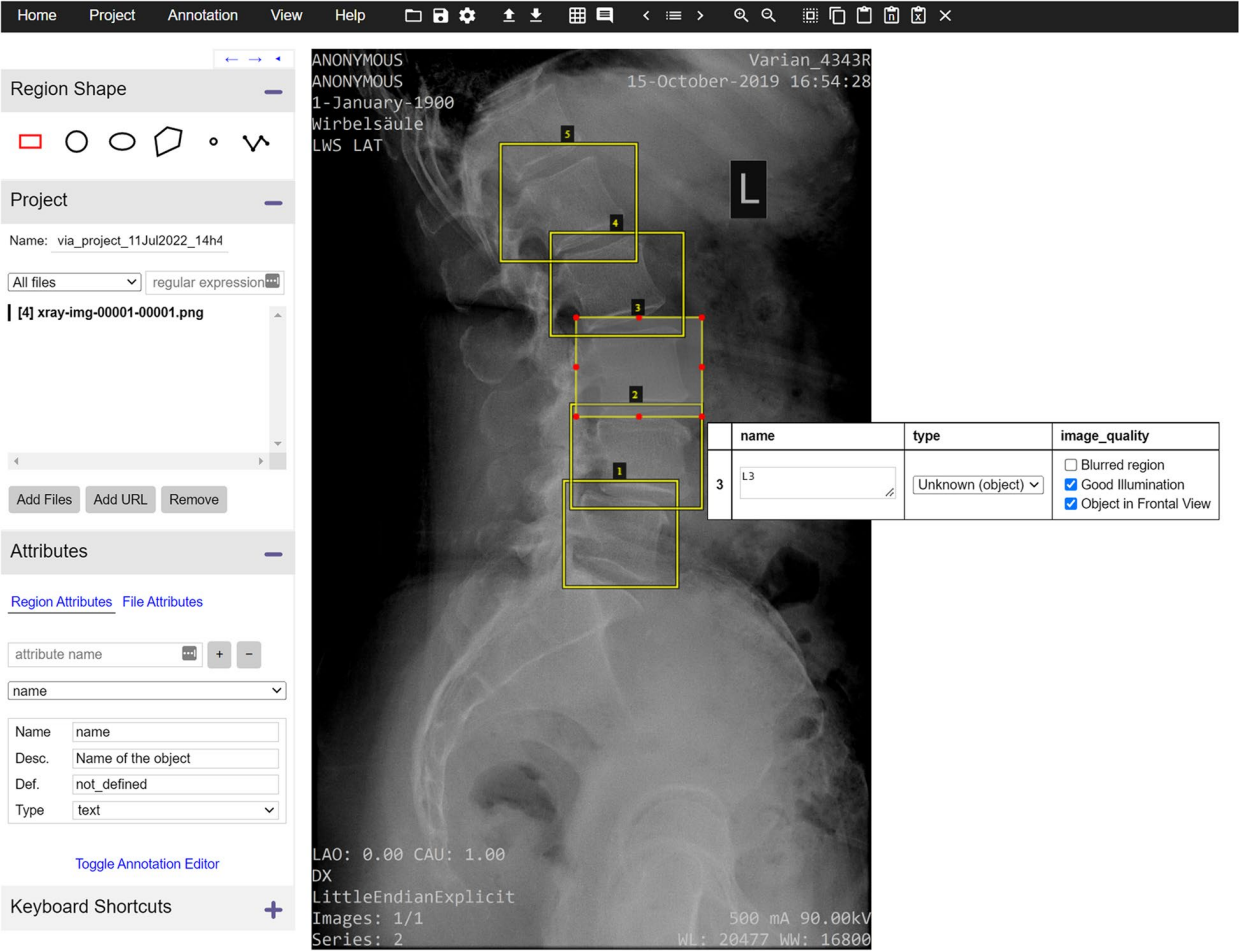
Screenshot of VGG Image Annotator, a free online platform for image annotation

While the standard image processing packages such as ImageJ and 3D Slicer are commonly used to generate image annotations, dedicated software allowing for a fast workflow has been developed and made available, both by the free software community and for commercial purposes. An example of a free image annotation tool is VGG Image Annotator [36] (https://www.robots.ox.ac.uk/~vgg/software/via), an online tool developed at the University of Oxford which allows drawing regions of interest by means of manual tools such as circles, rectangles, and polygons, to associate a name to each region and one or more customised values (Fig. 5). The annotation can then be downloaded as a text file in the commonly employed “Common Objects in Context” (COCO) format [37]. Another free online platform is MakeSense.AI (https://www.makesense.ai/), which offers similar functionality and is available through the GPLv3 licence. Intel Corporation (Santa Clara, CA, USA) also released an open-source tool, “Computer Vision Annotation Tool” (CVAT) which provides enhanced tools especially targeting video files (https://cvat.org/).

**Fig. 6:**
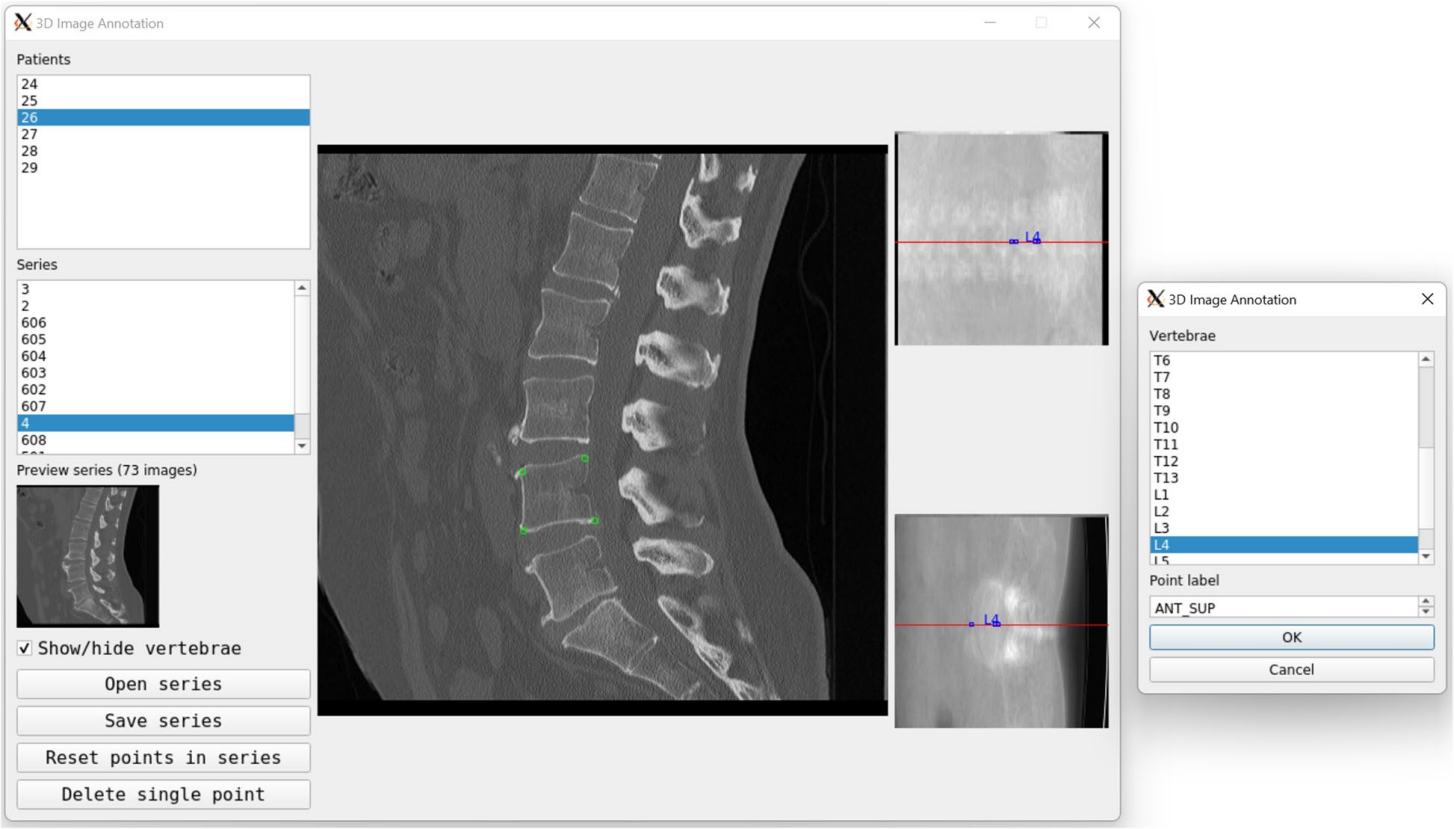
Custom image annotation software developed by the authors, aimed at localising landmarks (green circles) in 3D images of the spine. This Python application runs on a local computer and does not require sharing any information over the Internet. The user interface is designed to minimise any human interaction not directly aimed at localising the landmarks on the images, considerably speeding up the annotation workflow

While general-purpose image annotation software is gradually becoming dominant for the labelling of medical images, software purposely designed for a specific annotation task is still being developed, especially in the academic context (Fig. 6).

It should be noted that the use of such software has major advantages: it does not require uploading images to the Internet (which may not be allowed or can be restricted by some specific hospitals or by local regulations), and the user interface may be designed to take advantage of the characteristics of the data to further improve efficiency. On the other side, the development of annotation software requires time and human resources, and the program itself could not be reused in another project without extensive modifications.

Due to the high interest in AI-based computer vision for commercial applications, several non-free tools offering additional features are also available. Some examples of such tools include V7 (V7 Labs, London, UK, https://www.v7labs.com/), Labelbox (Labelbox, San Francisco, CA, https://labelbox.com/), and DataLoop (Dataloop, Herzliya, Israel, https://dataloop.ai/). These packages emphasise efficiency in the management of large datasets, the possibility of scripting and automatising pipelines, team working on the same datasets, and fast and simple integration with ML frameworks. Access to expert human labellers through the same user interface can also be offered, for example, by V7.

Vendors of PACS and medical imaging equipment are also offering on a commercial basis software that can be used for image annotation and preparation for AI development, although detailed documentation about such solutions is usually not made publicly available. Examples are Siemens Healthineers (Erlangen, Germany) with the “syngo.via” client-server platform, and Philips (Amsterdam, The Netherlands) with “IntelliSpace Discovery”. The latter framework provides access to the full process of data preparation, model training, and deployment in a research environment, including image annotation, advanced visualisation, tools for semi-automatic segmentation, and radiomics.

Image annotation software can be coupled with AI-based models to facilitate and accelerate the annotation itself. For example, pre-trained object detection models such as You Only Look Once (YOLO) [38] can be used to identify potential regions of interest within the image, leaving to the human annotator only the task of accepting, rejecting, or modifying them and therefore speeding up the whole process. Similarly, general-purpose segmentation models can be used to provide a pre-annotated image to the annotator, who may accept it or edit it if necessary. While such accelerating tools are mostly offered by commercial vendors (*e.g.*, V7 and Labelbox), some open-source packages such as the “Computer Vision Annotation Tool” also provide access to them. The practical advantage offered by these tools depends on the specific application, besides on the performance of the assisting models themselves; since most of them are designed to process photographs and web content and not medical imaging, their applicability to radiology needs to be proven on a case-specific basis.

## Curation and storage

Data curation involves all procedures aimed at ensuring that the database used for training and validation of the models has integrity, consistency, traceability, and finally high quality in general [22]. While annotation should be considered an integral part of data curation, other activities need to complement it in order to guarantee the quality of the collected data. Such activities include searching the database for inconsistencies and duplicates, detecting issues such as missing data and broken links, verifying the compliance of the images to the DICOM format, and finding images with insufficient quality, incorrect fields of view, or acquisition protocols different from those planned for the specific study.

While the methods used for data curation depend strongly on the specific project and cannot be easily generalised, software tools that can be useful in several cases are available. An example is POSDA (https://posda.com/), an open-source framework aimed at curating databases of DICOM files. Among its various features, POSDA can check the conformity of the image to the standard and detect images with the same unique identifier through either a user-friendly graphical interface or Perl scripting. Other open-source projects that offer similar functionalities include DVTk (https://www.dvtk.org/) and dicom3tools (https://www.dclunie.com/dicom3tools.html).

Another important part of data curation is *harmonisation* [35]. Harmonising medical images aims at ensuring that variables such as the scanner model, the magnetic field intensity for MRI, the spatial resolution, the reconstruction technique, and the acquisition parameters in general do not have a significant effect on the predictions of the AI-based tools to be developed. Various pre-processing techniques are employed for this purpose including *denoising*, *intensity normalisation*, and *removal of artefacts*. The choice of the most appropriate harmonising techniques strictly depends on the imaging modality; for example, intensity normalisation might not be needed in the case of quantitative imaging methods such as quantitative CT or T2 mapping in MRI.

The *intensity normalisation* could influence some tasks such as texture classification where the aim is to give meaning to pixels’ spatial variations that can provide useful insights into tissue structure. Collewet et al. [39] studied the effect of normalisation on MRI images by comparing different intensity normalisation techniques, namely original grey levels (no normalisation), same maximum for all images, same mean for all images, and dynamics limited to *μ* ± 3*σ*. They found that if the original grey levels are kept, the classification errors depend on the acquisition protocol.

Another work [40] also studied the impact of normalisation on texture classification. They studied the min-max, the 1−99%, and the 3*σ* normalisations and found that 1−99% normalisation is the best solution to normalise both CT and MRI images. The 1−99% normalisation simply saturates the bottom 1% and the top 1% of all pixel values to enhance image contrast. In [41], the authors investigated the standardisation of MRI images across different machines and protocols. They analysed three intensity normalisation methods (Nyul, WhiteStripe, *Z*-score) as well as two methods for intensity discretisation (fixed bin size and fixed bin number) to understand their impact on a tumour grade classification task (balanced accuracy measurement) using five well-established ML algorithms. The results showed that the mean balanced accuracy for tumour grade classification was increased from 0.67 to 0.82, 0.79 and 0.82 respectively using the Nyul, WhiteStripe, and *Z*-score normalisation methods compared to no normalisation.

Concerning X-ray images, Brahim et al. [42] proposed a novel normalisation technique based on a predictive modelling using multivariate linear regression to reduce the inter-subject variability in patients affected by osteoarthritis. This normalisation is preceded by a pre-processing step in the Fourier domain using a circular Fourier filter. Using random forests and naive Bayes classifiers, the authors achieved good results in terms of accuracy (82.98%), sensitivity (87.15%), and specificity (80.65%).

The curated database needs to be stored using technologies compliant with the criteria of security, integrity, and consistency as well [22]. A common solution is to use a PACS server, in most projects, the same is already in use in the hospital that is collecting the data. However, an independent PACS could be set up specifically for the project, commonly choosing from several open-source solutions such as Orthanc (https://www.orthanc-server.com/) and Dicoogle (https://dicoogle.com/). In recent years, the Extensible Neuroimaging Archive Toolkit (XNAT) platform (https://www.xnat.org/) is gaining a wider and wider user base, especially in academic and research environments. XNAT is compliant with the DICOM standard and privacy regulations but does not follow the PACS specifications, making it a more modern and flexible alternative which allows easier integration with advanced research software.

## Conclusions

Ensuring that high-quality images and annotations are used for the development and validation of AI-based algorithms in radiology has become a widely discussed topic in recent years, and its importance for achieving high accuracy and robustness in model predictions is nowadays universally acknowledged. As shown in this narrative review, several software tools are available either with open-source licences or commercially and can be very effective if used correctly. Local regulations regarding privacy, data handling and security, and compliance with standards must guide the definition of the image preparation workflow and must be considered when choosing the annotation and curation tools.

## Data Availability

There is no data associated with this paper.

## Abbreviations

2D: Two-dimensional
3D: Three-dimensional
AI: Artificial intelligence
CT: Computed tomography
DICOM: Digital Imaging and Communications in Medicine
GDPR: General Data Protection Regulation
HIPAA: Health Insurance Portability and Accountability Act
ITK: Insight Toolkit
ML: Machine learning
MRI: Magnetic resonance imaging
NIfTI: Neuroimaging Informatics Technology Initiative
PACS: Picture Archiving and Communication Systems
RGB: Red, green, blue
WSI: Whole-slide image
